# Nomogram Development and Feature Selection Strategy Comparison for Predicting Surgical Site Infection After Lower Extremity Fracture Surgery

**DOI:** 10.3390/medicina61081378

**Published:** 2025-07-30

**Authors:** Humam Baki, Atilla Sancar Parmaksızoğlu

**Affiliations:** 1 Department of Orthopedics, Private Gaziosmanpaşa Hospital, Istanbul Yeni Yüzyıl University, 34010 Istanbul, Türkiye; 2 Department of Orthopedics, Türkiye Hospital, 34381 İstanbul, Türkiye; asparmaksiz@yahoo.com

**Keywords:** surgical wound infection, fractures, bone, lower extremity, risk assessment, nomograms

## Abstract

*Background and Objectives*: Surgical site infections (SSIs) are a frequent complication after lower extremity fracture surgery, yet tools for individualized risk prediction remain limited. This study aimed to develop and internally validate a nomogram for individualized SSI risk prediction based on perioperative clinical parameters. *Materials and Methods*: This retrospective cohort study included adults who underwent lower extremity fracture surgery between 2022 and 2025 at a tertiary care center. Thirty candidate predictors were evaluated. Feature selection was performed using six strategies, and the final model was developed with logistic regression based on bootstrap inclusion frequency. Model performance was assessed by area under the curve, calibration slope, Brier score, sensitivity, and specificity. *Results*: Among 638 patients undergoing lower extremity fracture surgery, 76 (11.9%) developed SSIs. Of six feature selection strategies compared, bootstrap inclusion frequency identified seven predictors: red blood cell count, preoperative C-reactive protein, chronic kidney disease, operative time, chronic obstructive pulmonary disease, body mass index, and blood transfusion. The final model demonstrated an AUROC of 0.924 (95% CI, 0.876–0.973), a calibration slope of 1.03, and a Brier score of 0.0602. Sensitivity was 86.2% (95% CI, 69.4–94.5) and specificity was 89.5% (95% CI, 83.8–93.3). Chronic kidney disease (OR, 88.75; 95% CI, 5.51–1428.80) and blood transfusion (OR, 85.07; 95% CI, 11.69–619.09) were the strongest predictors of infection. *Conclusions*: The developed nomogram demonstrates strong predictive performance and may support personalized SSI risk assessment in patients undergoing lower extremity fracture surgery.

## 1. Introduction

Lower extremity fractures are among the most common orthopedic injuries requiring surgical treatment [1,2]. Fractures of the patella, tibia, fibula, and ankle are commonly reported and often necessitate operative management [3]. Surgical fixation remains the standard treatment approach to achieve anatomical realignment and restore function in these injuries [4,5]. However, surgical site infections (SSIs) remain a frequent complication after lower extremity fracture surgery, contributing to longer hospital stays, reoperations, and increased healthcare resource utilization [6]. SSI rates after lower extremity fracture surgery range from 3.6% in closed fractures to nearly 19% in open fractures, largely influenced by the severity of the fracture and surgical complexity [7,8]. Beyond their frequency, SSIs are associated with significant morbidity, including delayed wound healing, increased risk of nonunion, and the need for reoperations [9]. Early identification of patients at increased risk of SSI is a critical component of postoperative management.

Nomograms are widely used for individualized risk estimation in surgical populations, unlike traditional risk scoring systems that rely on categorical variables. By integrating continuous and categorical variables, they allow for patient-specific risk predictions in a visual format [10]. Multiple studies in orthopedic surgery have validated their utility for infection risk stratification and clinical decision-making [11,12,13]. The predictive accuracy of a nomogram depends on the appropriate selection of variables included in the model. Feature selection techniques, including univariate analysis, stepwise regression, and penalization methods, such as least absolute shrinkage and selection operator (LASSO), are commonly applied to minimize overfitting and improve model generalizability [14].

We developed and internally validated a nomogram to predict the risk of SSI following lower extremity fracture surgeries. Multiple feature selection strategies were compared to identify the approach providing optimal predictive accuracy, calibration, and clinical applicability.

## 2. Materials and Methods

### 2.1. Study Design and Setting

This retrospective cohort study was conducted at Istanbul Yeni Yüzyıl University Hospital, a tertiary care academic center. The study protocol was approved by the Institutional Review Board of Istanbul Yeni Yüzyıl University (approval number: 2025/05-1543; date: 7 May 2025). Due to the retrospective nature of the study, the requirement for informed consent was waived. This study was conducted and reported in accordance with the TRIPOD guidelines [15]. TRIPOD adherence was cross-verified using the official TRIPOD checklist prior to manuscript submission. Patient confidentiality was maintained through anonymization of data prior to analysis.

### 2.2. Participants

Adult patients aged 18 years or older who underwent surgical treatment for lower extremity fractures between January 2022 and January 2025 were eligible for inclusion. Patients were excluded if they had a pre-existing infection at the time of surgery, pathological fractures attributable to malignancy or metabolic bone disease, chronic immunosuppressive therapy, a history of solid organ transplantation, or a diagnosis of human immunodeficiency virus infection. Patients managed non-surgically, those with incomplete clinical data, or those without complete 90-day postoperative follow-up were also excluded. For patients who underwent multiple surgeries during the study period, only the first operative intervention was included. In patients with multiple concurrent fractures, the fracture associated with the longest operative time and highest surgical complexity, as determined by the need for advanced reconstructive techniques, was selected for analysis.

### 2.3. Data Collection and Review

All records extracted from the hospital information management system were supplemented by manual chart review to verify missing or ambiguous entries. All patient data were abstracted using a standardized data collection form developed prior to the study and reviewed independently by two researchers. Inter-reviewer agreement for eligibility and outcome assessment was quantified using Cohen’s kappa coefficient. In cases of disagreement, a third senior reviewer adjudicated to reach consensus. A random audit of 5% of manually reviewed charts was performed to confirm data accuracy.

### 2.4. Outcome Definition

The primary outcome was the occurrence of SSI within 90 days postoperatively. SSI was defined according to the Centers for Disease Control and Prevention criteria, encompassing superficial incisional, deep incisional, and organ/space infections [16]. Classification was based on documented clinical signs, microbiological culture results, and radiological findings available in the medical record.

### 2.5. Predictor Variables

Candidate predictors included demographic variables (age, sex, and body mass index), comorbidities (diabetes mellitus, hypertension, malignancy, smoking status, chronic kidney disease, chronic obstructive pulmonary disease, and American Society of Anesthesiologists [ASA] physical status classification), and surgical characteristics (emergency surgery, presence of open fracture, tourniquet use, time to surgery from admission, operative time, surgical technique [minimally invasive versus open], use of external fixation, flap coverage, bone grafting, drain insertion, reoperation, blood transfusion, estimated blood loss, and length of hospital stay). Standard perioperative antibiotic prophylaxis was administered in accordance with institutional guidelines. Laboratory measurements included hemoglobin, red blood cell count, white blood cell count, neutrophil count, lymphocyte count, platelet count, prothrombin time, activated partial thromboplastin time, serum albumin, glucose, D-dimer, and C-reactive protein (CRP). All continuous variables were analyzed in their original form without transformation or categorization. Non-linear associations between continuous predictors and outcome were assessed using locally weighted scatterplot smoothing (LOWESS) plots.

### 2.6. Sample Size Considerations

The minimum required sample size was estimated based on a target shrinkage factor of 0.90, an anticipated Nagelkerke R^2^ of 0.45, the inclusion of ten predictors, and an expected event prevalence of 11.9%. This calculation indicated that at least 334 patients and 40 outcome events were necessary. The final study sample comprised 638 patients, with 76 (11.9%) experiencing SSI, corresponding to an events-per-predictor (EPP) ratio of 7.6.

### 2.7. Missing Data

No variable had more than 5% missing data in the final dataset of 638 patients. Laboratory parameters demonstrated the highest rates of missingness, with serum albumin missing in 15 patients (2.3%), D-dimer in 20 patients (3.1%), preoperative CRP in 12 patients (1.9%), and postoperative CRP in 17 patients (2.7%). Demographic, comorbidity, and surgical variables were complete. Missing values were imputed using multiple imputation by chained equations. Predictive mean matching was applied for continuous variables and logistic regression imputation for categorical variables. Five imputed datasets were created. Convergence of imputation models was evaluated through inspection of trace plots. Missingness was assumed to be missing at random (MAR) and was assessed using Little’s MCAR test. The number of imputations was determined sufficient based on the fraction of missing information (FMI), which remained below 0.2 for all variables. Complete definitions of all candidate predictors, missingness rates, and imputation strategies are provided in Supplementary Table S1. All variables were included in the imputation models, and continuous predictors were used in their raw scale without normalization or transformation.

### 2.8. Nomogram Construction in Clinical Risk Prediction

Nomograms have emerged as practical tools in orthopedic and surgical risk prediction due to their ability to translate complex regression models into user-friendly visual formats. Unlike categorical risk scores, nomograms preserve the predictive value of continuous variables and allow individualized risk estimation. Their interpretability and bedside applicability have led to increasing adoption in orthopedic infection risk stratification, particularly for SSI [11,17].

In this study, we selected a nomogram approach to create a clinically applicable and visually interpretable model for estimating SSI risk following lower extremity fracture surgeries. This decision was based on the heterogeneity of relevant predictors—spanning demographic, surgical, and laboratory domains—which warranted an integrated multivariable modeling framework [18]. Furthermore, prior studies have demonstrated that nomograms provide reliable calibration and discrimination in orthopedic contexts when combined with modern validation tools such as bootstrap resampling and decision curve analysis [13].

### 2.9. Feature Selection Strategies in Prediction Model Development

Robust feature selection is essential for developing generalizable clinical prediction models. Given the wide range of variables potentially associated with SSI, we systematically compared six feature selection strategies to balance model performance with interpretability. These included representative methods from three main families: filter (univariate analysis), wrapper (stepwise selection, recursive feature elimination), and embedded approaches (LASSO, Boruta). Each method carries distinct advantages and limitations in terms of computational efficiency, variable collinearity handling, and modeling bias [19].

In high-dimensional clinical datasets, embedded and regularized approaches like LASSO have gained prominence due to their ability to prevent overfitting and simplify models [20]. However, wrapper methods such as recursive feature elimination often yield superior discrimination in well-structured datasets by accounting for interactions [21].

Given these considerations, we implemented multiple strategies and benchmarked them across discrimination (AUROC), calibration, and decision metrics. This comparative approach has been previously applied in orthopedic infection modeling with favorable results, and is recommended for achieving balance between model complexity and predictive power [22,23]. Thresholds for variable retention and multicollinearity control strategies for each selection method are summarized in Supplementary Table S2.

### 2.10. Analysis

All statistical analyses were performed using R version 4.4.2 (R Foundation for Statistical Computing, Vienna, Austria). Continuous variables were assessed for normality using histogram visualization. Normally distributed variables were reported as mean ± standard deviation, and compared using independent samples t-tests. Non-normally distributed variables were summarized as median [interquartile range] and compared using the Mann–Whitney U test. Categorical variables were expressed as counts and percentages (*n* [%]) and compared using the Chi-square or Fisher’s exact test as appropriate.

Feature selection was performed using six different strategies: bootstrap inclusion frequency, LASSO, univariate filtering (*p* < 0.20), stepwise selection, Boruta algorithm, and recursive feature elimination (RFE). Models derived from each selection method were evaluated using logistic regression or Firth’s penalized logistic regression in the presence of separation or convergence instability.

Model performance was assessed in a 70/30 train-test split using multiple metrics: area under the receiver operating characteristic curve (AUROC), Brier score, calibration slope, Akaike information criterion (AIC), and Bayesian information criterion (BIC). For models with missing calibration statistics or failed convergence, diagnostic limitations were noted. The final model was selected based on a balance of discrimination, calibration, parsimony, and clinical interpretability. Odds ratios (ORs) with 95% confidence intervals (CI) were reported for all predictors in the final model. A nomogram was constructed using the rms package based on this model.

Model development adequacy was assessed using the pmsampsize package. Given a Nagelkerke R^2^ of 0.71 and 8 predictors, the minimum required sample size was estimated as 243 with at least 29 events, assuming a target shrinkage of 0.9. Our final sample (*n* = 638; events = 76) exceeded these thresholds, corresponding to an EPP of 9.5, supporting model stability and low overfitting risk.

## 3. Results

A total of 638 patients undergoing lower extremity fracture surgery were included, of whom 76 (11.9%) developed SSI. Inter-reviewer agreement for eligibility assessment yielded a Cohen’s kappa coefficient of 0.84 (95% CI, 0.78–0.90). For outcome adjudication of SSIs, the Cohen’s kappa coefficient was 0.81 (95% CI, 0.74–0.88). Disagreements occurred in 18 cases (2.8%) for eligibility assessment and 12 cases (1.9%) for outcome adjudication. All disagreements were resolved through consensus by a third senior reviewer.

Comparative characteristics between the SSI and non-SSI groups are presented in Table 1 and Table 2. The SSI group had higher BMI, longer time to surgery, greater operative time, higher transfusion and flap coverage rates, and elevated inflammatory markers such as CRP, D-dimer, and neutrophil count (all *p* < 0.05). Lymphocyte count and albumin levels were significantly lower in the SSI group.

Feature selection and model building were performed using six distinct strategies (Table 3). During this process, several methodological issues emerged. In the LASSO-based model, 21 candidate variables were initially identified, but the final penalized solution retained only one predictor. This sparse configuration resulted in near-complete separation, with a maximum predicted probability of 0.997 for the positive class in the training set and a convergence warning flag during model fitting. Penalized logistic regression with Firth correction was applied to mitigate this issue and ensure coefficient stability, but performance remained suboptimal, with reduced precision in cross-validation and extreme calibration behavior.

In the Boruta-based selection strategy, the algorithm yielded 16 predictors with high selection frequency, but several of these showed multicollinearity and unstable estimates when entered into a traditional logistic model. To stabilize coefficient shrinkage and address variance inflation, ridge penalization was applied in the final logistic regression step, improving the condition number from 47.2 to 9.5. However, this adjustment led to inflated slope values and highly optimistic AUROC estimates in the test set.

Univariate filtering allowed the inclusion of 27 variables, 7 of which had a positive class frequency below 5%. Although the Univariate + Firth model achieved an AUROC of 0.997 in the test set, its predictive reliability was undermined by overly sparse inputs. Moreover, due to complete separation and degenerate predictions in the minority class, sensitivity and specificity values could not be reliably reported.

The RFE + LRM model retained 13 variables and yielded excellent discrimination, but failed to produce a valid Hosmer–Lemeshow test statistic due to the convergence instability observed in repeated validation folds. The stepwise model showed a similar pattern of calibration weakness despite acceptable AUROC.

**Figure 1:**
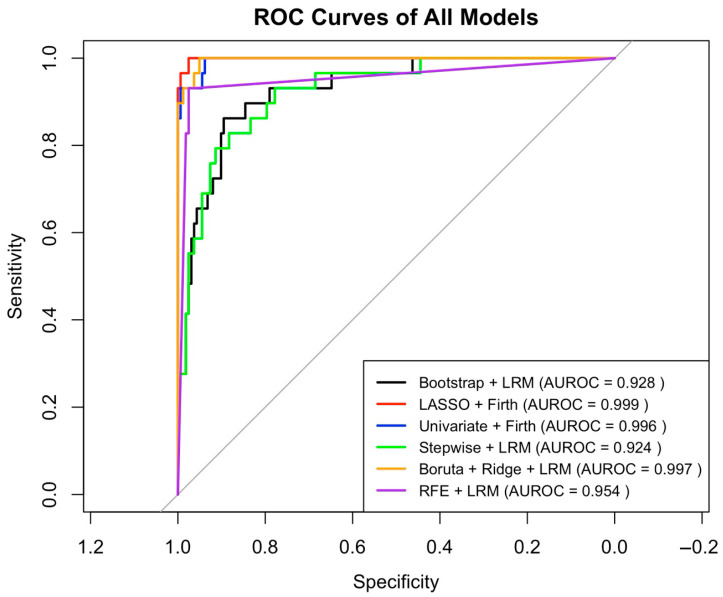
ROC curves of all models for SSI prediction. ROC curves of six prediction models for surgical site infection. AUROC values are presented in the legend. The diagonal line represents the reference line (no discrimination).

A comparative evaluation of all models in the test set is presented in Table 4. While some models achieved extremely high AUROC values (e.g., Univariate + Firth: 0.997; Boruta + Ridge: 0.997), they also displayed clear signs of overfitting. These included either extremely low ΔAUROC values (<0.005) with high calibration slope (>5.0), or exceptionally low Brier scores coupled with implausibly confident predictions. For instance, the Boruta + Ridge + LRM model had the lowest AIC (53.49), yet its slope exceeded 8.1, reflecting poor generalizability. Conversely, the Bootstrap + LRM model demonstrated a more stable profile with strong AUROC (0.924), acceptable ΔAUROC (0.0571), a reasonable Brier score (0.0602), and the best overall balance in AIC (114.62) and BIC (151.54) among all non-penalized models. The ROC curves of all candidate models are shown in Figure 1.

**Figure 2:**
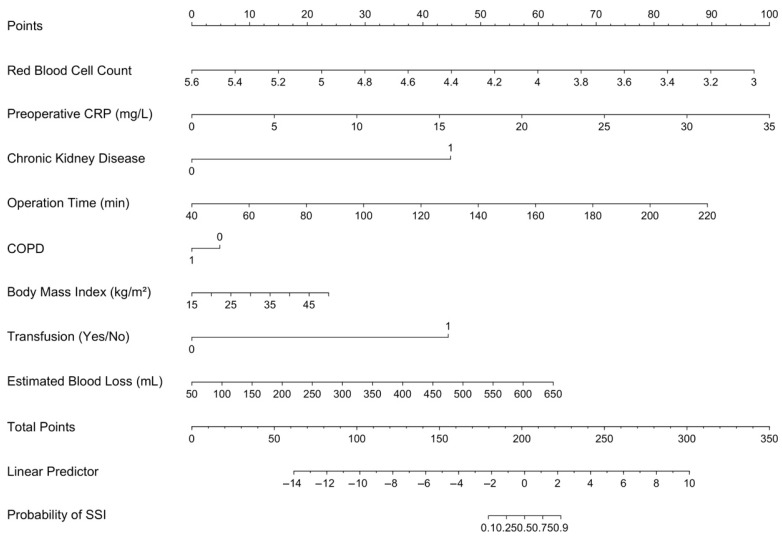
Nomogram derived from the final prediction model. Nomogram developed using the final logistic regression model (Bootstrap + LRM) to predict risk of surgical site infection. Point assignments for each variable are shown at the top, with the total score mapped to predicted probability at the bottom.

Taken together, Bootstrap + LRM offered the most interpretable and well-calibrated solution, with no convergence issues, no implausible confidence shifts, and a manageable variable count (*n* = 7). This model was selected for final implementation and nomogram construction. This final model incorporated seven predictors. The adjusted ORs for these predictors are presented in Table 5. The resulting nomogram is illustrated in Figure 2, depicting the point contribution of each covariate and the corresponding total risk estimate for SSI. Although chronic obstructive pulmonary disease did not reach statistical significance, it was retained in the final model due to its consistent selection across bootstrap replicates and negligible impact on model calibration or discrimination.

## 4. Discussion

This study developed and internally validated a multivariable prediction model for SSI following lower extremity fracture surgery in a cohort of 638 patients, among whom 76 (11.9%) developed infection. A total of 29 candidate predictors were assessed using six feature selection strategies to identify a parsimonious model. The final model was incorporated into a nomogram to enable individualized risk estimation using routinely collected perioperative data.

Feature selection in clinical prediction modeling not only affects statistical performance but also determines how applicable a model is in real-world settings. In orthopedic trauma surgery, incorporating routinely available predictors such as CRP, blood transfusion, and chronic kidney disease ensures both usability and relevance. While approaches like LASSO and RFE demonstrated strong discrimination, they also showed convergence or calibration issues consistent with prior reports [24,25,26]. We therefore adopted bootstrap inclusion frequency, which preserved clinical interpretability, enhanced model stability, and reduced overfitting risk [27].

Reliable individualized risk prediction is essential for informing perioperative decision-making. In this study, the final model demonstrated strong discrimination, with an AUROC of 0.924, demonstrating strong separation between patients who did and did not develop SSI. The observed discrimination level is comparable to that of the Ex-Care BR surgical risk model (AUROC = 0.93) and exceeds that observed in models predicting postoperative complications after ankle arthrodesis (AUROC approximately 0.71) [28,29]. Calibration was similarly robust, with a calibration slope of 1.03 and a Brier score of 0.0602, indicating low prediction error. These figures compare favorably with those reported for the ACS-NSQIP calculator in thoracic surgery (AUROC = 0.67; Brier score > 0.10) and models predicting perioperative complications in spine surgery (Brier score approximately 0.094) [30,31]. Together, the model’s discrimination, calibration, and low prediction error highlight its potential role in perioperative risk assessment, where accurate individualized predictions are essential for guiding clinical decision-making [32].

While discrimination and calibration define overall model performance, clinical utility also depends on identifying predictors with strong, interpretable effects. In the final model, red blood cell count, preoperative CRP, chronic kidney disease, operative time, body mass index, blood transfusion, and estimated blood loss were selected based on bootstrap inclusion frequency. Several predictors demonstrated notably high odds ratios, including chronic kidney disease (OR, 88.75; 95% CI, 5.51–1428.80) and blood transfusion (OR, 85.07; 95% CI, 11.69–619.09), reinforcing their established association with SSI risk. While bootstrap-based selection improves robustness, these large effect sizes may also reflect residual confounding or the influence of small subgroup counts and should be interpreted with caution. Chronic kidney disease has been identified as a significant risk factor for SSI in fracture surgeries, particularly in vascular procedures involving the lower limbs [33]. Operative time and estimated blood loss also showed strong associations, consistent with prior studies where prolonged surgery and higher intraoperative blood loss were independently linked to increased SSI rates in orthopedic trauma patients [34]. Similarly, blood transfusion has been repeatedly cited as a modifiable risk factor for SSIs in lower extremity fracture surgery [35]. The model’s sensitivity (86.2%) and specificity (89.5%) further reflect its predictive strength. Comparable machine learning models predicting SSI after lower extremity fracture surgeries have reported AUROCs around 0.78 with lower sensitivity and specificity values, highlighting the relative improvement in our model’s performance [34]. These metrics suggest a well-calibrated balance: minimizing false negatives—crucial in infection prevention—while avoiding unnecessary interventions for low-risk patients. The consistency with established risk factors and the strong predictive ability support the integration of this model into individualized perioperative care pathways for lower extremity fracture surgery. Recent machine learning models for SSI prediction have reported comparable AUROC values, but their limited interpretability and lack of clinical transparency reduce their bedside applicability [34,35].

Translating individualized risk estimates into clinical practice offers the potential to refine perioperative strategies in orthopedic surgery. Identifying patients at increased risk for SSI may support more targeted preventive efforts and patient-centered decision-making. The deliberate comparison of feature selection methods emphasizes the importance of balancing model complexity with clinical usability. Future research could examine how risk models integrate into perioperative workflows and assess their impact on patient outcomes. However, implementation of this nomogram into clinical practice would require additional steps, including integration with electronic health records, clinician training, and decision support system validation. Moreover, patient-specific factors and institutional protocols may affect generalizability despite the model’s strong internal performance.

### Limitations

This study has several limitations. First, its retrospective design may affect the completeness and consistency of data capture, despite strict eligibility criteria and independent outcome adjudication. Second, the analysis was conducted at a single tertiary care center, which may limit generalizability to broader or more diverse populations. Third, although multiple imputation addressed missing data, the possibility of unmeasured confounding cannot be excluded. Fourth, while multiple feature selection strategies were systematically compared, other modeling approaches not evaluated here might yield different results. Similarly, variables not selected or included in the final model, despite their potential relevance, may contribute to SSI risk in other contexts. Fifth, external validation in independent cohorts is necessary to confirm the model’s performance. Future studies may consider prospective multicenter designs or leverage national orthopedic registry data to evaluate generalizability across different practice settings and patient populations.

## 5. Conclusions

This study developed a nomogram to predict SSI after lower extremity fracture surgery based on routinely collected perioperative variables. The final model incorporated red blood cell count, preoperative CRP, chronic kidney disease, operative time, body mass index, blood transfusion, and estimated blood loss. The nomogram enables the translation of routinely collected perioperative data into individualized SSI risk estimates. Further validation is necessary to establish its generalizability.

## Figures and Tables

**Table 1:** Baseline demographics, clinical characteristics, and laboratory findings according to surgical site infection status.

Variable	SSI− (*n* = 562)	SSI+ (*n* = 76)	*p*	Mean Difference (95% CI)
Age (years)	48.1 ± 18.7	52.8 ± 18.7	0.045	4.64 (0.12 to 9.17)
Sex (Male)	348 (61.9%)	54 (71.1%)	0.155	
BMI (kg/m^2^)	26.9 ± 9.5	30.1 ± 10.6	0.015	3.18 (0.64 to 5.71)
Diabetes mellitus	65 (11.6%)	19 (25.0%)	0.002	
Hypertension	183 (32.6%)	34 (44.7%)	0.048	
Malignancy	16 (2.8%)	6 (7.9%)	0.054	
Smoking	117 (20.8%)	15 (19.7%)	0.946	
CKD	20 (3.6%)	5 (6.6%)	0.338	
COPD	53 (9.4%)	10 (13.2%)	0.414	
ASA Score = 3–4	18 (3.2%)	5 (6.6%)	0.174	
Emergency surgery	172 (30.6%)	30 (39.5%)	0.153	
Open fracture present	78 (13.9%)	26 (34.2%)	<0.001	
Tourniquet use	386 (68.7%)	33 (43.4%)	<0.001	
Time to surgery (hours)	17.4 ± 7.3	22.3 ± 9.2	<0.001	4.87 (2.69 to 7.05)
OR time (minutes)	111.7 ± 26.8	126.0 ± 27.0	<0.001	14.29 (7.73 to 20.84)
Minimally invasive technique	133 (23.7%)	7 (9.2%)	0.007	
Fixation: external fixator	135 (24.0%)	32 (42.1%)	0.005	
Flap coverage performed	3 (0.5%)	18 (23.7%)	<0.001	
Bone grafting performed	0 (0.0%)	10 (13.2%)	<0.001	
Drain inserted	223 (39.7%)	43 (56.6%)	0.007	
Reoperation	2 (0.4%)	9 (11.8%)	<0.001	
Blood transfusion	18 (3.2%)	20 (26.3%)	<0.001	
Estimated blood loss (mL)	235.0 ± 112.6	302.2 ± 126.9	<0.001	67.15 (36.75 to 97.56)
Length of hospital stay (days)	4.4 ± 2.1	8.9 ± 4.8	<0.001	4.53 (3.42 to 5.64)

ASA: American Society of Anesthesiologists; CKD: chronic kidney disease; COPD: chronic obstructive pulmonary disease; OR: operating room; SSI: surgical site infection.

**Table 2:** Laboratory findings according to surgical site infection status.

Variable	SSI− (*n* = 562)	SSI+ (*n* = 76)	*p*	Mean Difference (95% CI)
Hemoglobin (g/dL)	130.7 ± 14.1	129.7 ± 14.6	0.571	
RBC count (×10^6^/μL)	4.4 ± 0.4	4.3 ± 0.4	0.009	–0.14 (–0.24 to –0.04)
WBC count (×10^9^/L)	10.0 ± 1.7	10.5 ± 1.7	0.011	0.55 (0.13 to 0.98)
Neutrophil count (×10^9^/L)	6.2 ± 1.2	6.9 ± 1.2	<0.001	0.74 (0.44 to 1.03)
Lymphocyte count (×10^9^/L)	1.5 ± 0.3	1.3 ± 0.3	<0.001	–0.24 (–0.31 to –0.17)
Platelet count (×10^3^/μL)	239.0 ± 44.5	240.7 ± 50.2	0.780	
Prothrombin time (s)	11.0 ± 0.6	11.0 ± 0.6	0.476	
APTT (s)	25.9 ± 1.8	26.1 ± 2.0	0.473	
Albumin (g/dL)	40.6 ± 1.8	39.8 ± 1.5	<0.001	–0.79 (–1.17 to –0.41)
Glucose (mg/dL)	125.6 ± 23.2	151.6 ± 28.7	<0.001	26.03 (19.22 to 32.85)
D-Dimer (mg/L)	6.9 ± 4.2	15.1 ± 9.1	<0.001	8.13 (6.03 to 10.22)
Preoperative CRP (mg/L)	10.1 ± 6.0	17.7 ± 6.5	<0.001	7.62 (6.06 to 9.19)
Postoperative CRP (mg/L)	74.5 ± 32.1	115.1 ± 33.7	<0.001	40.68 (32.55 to 48.81)

APTT: activated partial thromboplastin time; CRP: C-reactive protein; D-Dimer: D-dimer fibrin degradation product; RBC: red blood cell; WBC: white blood cell; SSI: surgical site infection.

**Table 3:** Ranked performance of robust models by AUC and 95% confidence intervals.

Feature Selection Method	Variables Selected by FS (*n*)	Variables in Final Model (*n*)	AUROC (95% CI)	Sensitivity (95% CI)	Specificity (95% CI)	Hosmer–Lemeshow *p*	Nagelkerke R^2^
Bootstrap + LRM	9	7	0.924 (0.876–0.973)	0.862 (0.694–0.945)	0.895 (0.838–0.933)	0.367	0.708
LASSO + Firth	21	1	0.755 (0.657–0.852)	0.724 (0.543–0.853)	0.691 (0.616–0.757)	0.0002	0.310
Univariate + Firth (Shrinked)	27	27	0.997 (0.989–1.000)	Not reportable	Not reportable	0.118	1.00 (pre-shrink)
Stepwise + LRM	18	12	0.882 (0.814–0.950)	0.414 (0.255–0.593)	0.963 (0.922–0.983)	<0.001	0.664
Boruta + Ridge + LRM	16	9	0.997 (0.992–1.000)	0.931 (0.780–0.981)	0.988 (0.956–0.997)	1.000	0.934
RFE + LRM	13	13	0.954 (0.905–1.000)	0.897 (0.736–0.964)	0.975 (0.938–0.990)	Not available	1.000

LRM: Logistic Regression Model; FS: feature selection; AUROC: area under the receiver operating characteristic curve; CI: confidence interval; R^2^: Nagelkerke’s Pseudo R-squared; RFE: recursive feature elimination; Firth: Firth’s penalized logistic regression; Pre-shrink: Pre-penalization model performance; Not reportable: Performance metrics could not be computed due to complete separation or lack of predicted variability.

**Table 4:** Comparative diagnostic performance of models for SSI prediction in the test set.

Model	ΔAUROC	Brier Score	Calibration Slope	AIC	BIC
Bootstrap + LRM	0.0571	0.0602	3.2659	114.62	151.54
LASSO + Firth	0.1810	0.0117	12.6590	239.42	310.97
Univariate + Firth (Shrinked)	0.0030	0.0207	0.5343	199.59	327.87
Stepwise + LRM	0.0887	0.0772	5.7881	128.21	165.13
Boruta + Ridge + LRM	0.0019	0.0191	8.1792	53.49	106.82
RFE + LRM	0.0460	0.0366	5.8358	28.00	85.44

AUROC: area under the receiver operating characteristic curve; ΔAUROC: difference between training and test AUROC; Brier Score: mean squared error of predicted probabilities; Calibration Slope: logistic regression slope of predicted probabilities against actual outcome; AIC: Akaike information criterion; BIC: Bayesian information criterion.

**Table 5:** Adjusted odds ratios from the final logistic regression mode.

Variable	OR (95% CI)	*p*
RBC (×10^6^/μL)	0.13 (0.05–0.32)	<0.0001
Preoperative CRP (mg/L)	13.13 (5.18–33.30)	<0.0001
Chronic Kidney Disease	88.75 (5.51–1428.80)	0.0016
Operation Time (minutes)	5.41 (2.66–10.98)	<0.0001
COPD *	0.62 (0.11–3.41)	0.500
Body Mass Index (kg/m^2^)	3.06 (1.08–8.70)	0.036
Transfusion (Yes/No)	85.07 (11.69–619.09)	<0.0001
Estimated Blood Loss (mL)	5.37 (2.45–11.77)	<0.0001
Body Mass Index (kg/m^2^)	3.06 (1.08–8.70)	0.036

RBC: red blood cell count; CRP: C-reactive protein; COPD: chronic obstructive pulmonary disease; OR: odds ratio; CI: confidence interval. * COPD was retained in the final model and nomogram despite not reaching statistical significance (*p* = 0.50), as it was included in the bootstrap-selected variable set and did not adversely affect overall model performance.

## Data Availability

The data that support the findings of this study are available from the corresponding author, H.B., upon reasonable request.

## Supplementary Materials

The following supporting information can be downloaded at: https://www.mdpi.com/article/10.3390/medicina61081378/s1, Table S1: Variable Definitions and Imputation Strategy; Table S2: Feature Selection Thresholds and Multicollinearity Handling.

## Abbreviations

The following abbreviations are used in this manuscript:

SSI	Surgical Site Infection
AUROC	Area Under the Receiver Operating Characteristic Curve
CRP	C-Reactive Protein
BMI	Body Mass Index
ASA	American Society of Anesthesiologists
LASSO	Least Absolute Shrinkage and Selection Operator
RFE	Recursive Feature Elimination
AIC	Akaike Information Criterion
BIC	Bayesian Information Criterion
CDC	Centers for Disease Control and Prevention
LOWESS	Locally Weighted Scatterplot Smoothing
MAR	Missing at Random
MCAR	Missing Completely at Random
FMI	Fraction of Missing Information
EPP	Events-Per-Predictor
OR	Odds Ratio
CI	Confidence Interval

## References

[1] Zhang J., Bradshaw F., Hussain I., Karamatzanis I., Duchniewicz M., Krkovic M. (2024). The Epidemiology of Lower Limb Fractures: A Major United Kingdom (UK) Trauma Centre Study. Cureus.

[2] Beerekamp M.S.H., Keizer R.J.O.d.M., Schep N.W.L., Ubbink D.T., Panneman M.J.M., Goslings J.C. (2017). Epidemiology of Extremity Fractures in the Netherlands. Injury.

[3] Wu A.-M., Bisignano C., James S.L., Abady G.G., Abedi A., Abu-Gharbieh E., Alhassan R.K., Alipour V., Arabloo J., Asaad M. (2021). Global, Regional, and National Burden of Bone Fractures in 204 Countries and Territories, 1990–2019: A Systematic Analysis from the Global Burden of Disease Study 2019. Lancet Healthy Longev..

[4] Pigeolet M., Sana H., Askew M.R., Jaswal S., Ortega P.F., Bradley S.R., Shah A., Mita C., Corlew D.S., Saeed A. (2024). Outcomes of External versus Internal Fixation for Traumatic Lower Limb Fractures in Low- and Middle-Income Countries: A Systematic Review and Meta-Analysis Protocol. Bone Jt. Open.

[5] Chokotho L., Wu H.-H., Shearer D., Lau B.C., Mkandawire N., Gjertsen J.-E., Hallan G., Young S. (2020). Outcome at 1 Year in Patients with Femoral Shaft Fractures Treated with Intramedullary Nailing or Skeletal Traction in a Low-Income Country: A Prospective Observational Study of 187 Patients in Malawi. Acta Orthop..

[6] Parker B., Petrou S., Masters J.P.M., Achana F., Costa M.L. (2018). Economic Outcomes Associated with Deep Surgical Site Infection in Patients with an Open Fracture of the Lower Limb. Bone Jt. J..

[7] Liu H., Wang Y., Xing H., Chang Z., Pan J. (2024). Risk factors for deep surgical site infections following orthopedic trauma surgery: A meta-analysis and systematic review. J. Orthop. Surg. Res..

[8] Bai Y., Zhang X., Tian Y., Tian D., Zhang B. (2019). Incidence of Surgical-Site Infection Following Open Reduction and Internal Fixation of a Distal Femur Fracture: An Observational Case–Control Study. Medicine.

[9] Willey M., Karam M. (2016). Impact of Infection on Fracture Fixation. Orthop. Clin. N. Am..

[10] Li J., Xie X., Zhang J., Shen P., Zhang Y., Chen C., Si Y., Zou J. (2022). Novel Bedside Dynamic Nomograms to Predict the Probability of Postoperative Cognitive Dysfunction in Elderly Patients Undergoing Noncardiac Surgery: A Retrospective Study. CIA.

[11] Huang X., Guo Y., Fu R., Li H. (2023). A Nomogram to Predict Postoperative Surgical Site Infection of Adult Patients Who Received Orthopaedic Surgery: A Retrospective Study. Sci. Rep..

[12] Campbell M.P., Mott M.D., Owen J.R., Reznicek J.E., Beck C.A., Muthukrishnan G., Golladay G.J., Kates S.L. (2022). Low Albumin Level Is More Strongly Associated with Adverse Outcomes and Staphylococcus Aureus Infection than Hemoglobin A1C or Smoking Tobacco. J. Orthop. Res..

[13] An Y., Cui X., Wang H., Sun Y., Zhu B., Feng S., Jiang J. (2024). Nomogram for Predicting Surgical Site Infections in Elderly Patients after Open Lumbar Spine Surgery: A Retrospective Study. Int. Wound J..

[14] McNeish D.M. (2015). Using Lasso for Predictor Selection and to Assuage Overfitting: A Method Long Overlooked in Behavioral Sciences. Multivar. Behav. Res..

[15] Collins G.S., Moons K.G.M., Dhiman P., Riley R.D., Beam A.L., Van Calster B., Ghassemi M., Liu X., Reitsma J.B., van Smeden M. (2024). TRIPOD+AI Statement: Updated Guidance for Reporting Clinical Prediction Models That Use Regression or Machine Learning Methods. BMJ.

[16] Centers for Disease Control and Prevention, National Healthcare Safety Network (NHSN) (2024). Patient Safety Component Manual: Chapter 9—Surgical Site Infection (SSI) Event.

[17] Vonzun L., Rüegg L., Zepf J., Moehrlen U., Meuli M., Ochsenbein-Kölble N. (2023). Are Cervical Length and Fibronectin Predictors of Preterm Birth after Fetal Spina Bifida Repair? A Single Center Cohort Study. J. Clin. Med..

[18] Ma R., He J., Xu B., Zhao C., Zhang Y., Li X., Sun S., Zhang Q. (2020). Nomogram Prediction of Surgical Site Infection of HIV-Infected Patients Following Orthopedic Surgery: A Retrospective Study. BMC Infect. Dis..

[19] Liu H., Zhang W., Zhang Y., Zhang S., Jin G., Li X. (2023). Establishment and Validation of a Nomogram Model for Postoperative Surgical Site Infection after Transforaminal Lumbar Interbody Fusion: A Retrospective Observational Study. Surgery.

[20] Chen L., Liu C., Ye Z., Huang S., Liang T., Li H., Chen J., Chen W., Guo H., Chen T. (2022). Predicting Surgical Site Infection Risk after Spinal Tuberculosis Surgery: Development and Validation of a Nomogram. Surg. Infect..

[21] Liu H., Zhang W., Hu Q., Liu L., Xie Z., Xu Y., Jing G., Wang Y. (2023). A Nomogram for Accurately Predicting the Surgical Site Infection Following Transforaminal Lumbar Interbody Fusion in Type 2 Diabetes Patients, Based on Glycemic Variability. Int. Wound J..

[22] Cheng Y., Chen Y., Hou X., Yu J., Wen H., Dai J., Zheng Y. (2022). Development of a Nomogram for Predicting Surgical Site Infection in Patients with Resected Lung Neoplasm Undergoing Minimally Invasive Surgery. Surg. Infect..

[23] Luo J.-Z., Lin J.-Z., Chen Q.-F., Yang C.-J., Zhou C.-S. (2025). Construction and Validation of a Nomogram Predictive Model for Assessing the Risk of Surgical Site Infections Following Posterior Lumbar Fusion Surgery. Sci. Rep..

[24] Staartjes V.E., Kernbach J.M., Stumpo V., van Niftrik C.H.B., Serra C., Regli L., Staartjes V.E., Regli L., Serra C. (2022). Foundations of Feature Selection in Clinical Prediction Modeling. Machine Learning in Clinical Neuroscience.

[25] Xv Y., Lv F., Guo H., Liu Z., Luo D., Liu J., Gou X., He W., Xiao M., Zheng Y. (2021). A CT-Based Radiomics Nomogram Integrated With Clinic-Radiological Features for Preoperatively Predicting WHO/ISUP Grade of Clear Cell Renal Cell Carcinoma. Front. Oncol..

[26] Hamada M., Tanimu J.J., Hassan M., Kakudi H.A., Robert P. Evaluation of Recursive Feature Elimination and LASSO Regularization-Based Optimized Feature Selection Approaches for Cervical Cancer Prediction. Proceedings of the 2021 IEEE 14th International Symposium on Embedded Multicore/Many-core Systems-on-Chip (MCSoC).

[27] Lenert M.C., Walsh C.G. (2018). Balancing Performance and Interpretability: Selecting Features with Bootstrapped Ridge Regression. AMIA Annu. Symp. Proc..

[28] Passos S.C., Castro S.M.d.J., Stahlschmidt A., Neto P.C.d.S., Pereira P.J.I., Leal P.d.C., Lopes M.B., Falcão L.F.d.R., de Azevedo V.L.F., Lineburger E.B. (2024). Development and Validation of the Ex-Care BR Model: A Multicentre Initiative for Identifying Brazilian Surgical Patients at Risk of 30-Day in-Hospital Mortality. Br. J. Anaesth..

[29] Bugarin A., Shah A.A., Devana S., Lee C., SooHoo N.F. (2022). Development of a Machine Learning Algorithm for Prediction of Complications after Ankle Arthrodesis. Foot Ankle Orthop..

[30] Tsvetkov N., Mallaev M., Gahl B., Hojski A., Tamm M., Steiner L.A., Lardinois D. (2024). Validation of the American College of Surgeons Surgical Risk Calculator for Thoracic Surgery. J. Thorac. Dis..

[31] Shah A.A., Devana S., Lee C., Bugarin A., Upfill-Brown A., Lord E.L., Park D.Y., SooHoo N. (2021). P30. Development of a Machine Learning Algorithm for Prediction of Complications and Readmission after Lumbar Spinal Fusion. Spine J..

[32] Murray I., Lim K., Howells R., Jones R., Sharma A., Jones S. (2020). The Utility of a Personalised Risk Calculator in Gynae-Oncology Surgery. Clin. Oncol. Res..

[33] Davis F.M., Sutzko D.C., Grey S.F., Mansour M.A., Jain K.M., Nypaver T.J., Gaborek G., Henke P.K. (2017). Predictors of Surgical Site Infection after Open Lower Extremity Revascularization. J. Vasc. Surg..

[34] Gutierrez-Naranjo J.M., Moreira A., Valero-Moreno E., Bullock T.S., Ogden L.A., Zelle B.A. (2024). A Machine Learning Model to Predict Surgical Site Infection after Surgery of Lower Extremity Fractures. Int. Orthop. (SICOT).

[35] Sato T., Shibahashi K., Aoki M., Kudo D., Kushimoto S. (2024). Risk Factors for Surgical Site Infection Following Orthopaedic Surgery for Fracture by Trauma: A Nested Case–Control Study. J. Hosp. Infect..

