# Supplementary material for: Nomogram Development and Feature Selection Strategy Comparison for Predicting Surgical Site Infection After Lower Extremity Fracture Surgery
Source: Medicina (Kaunas). 2025 Jul 30;61(8):1378. doi: 10.3390/medicina61081378 (PMC12387606; doi:10.3390/medicina61081378)
Supplement: Supplementary file 1 [file medicina-61-01378-s001.zip › medicina-3730232-supplementary.pdf]

Supplementary Table 1. Variable Definitions and Imputation Strategy

**Variable**	**Definition**	**Missing Rate (%)**	**Imputation Method**
Age (years)	Age at time of surgery, in years	0.0%	Not applicable
Sex	Biological sex (Male/Female) as recorded in the electronic medical record	0.0%	Not applicable
Body Mass Index (kg/m^2)	Weight (kg) / Height² (m^2); measured at preoperative assessment	0.3%	Predictive Mean Matching
Diabetes Mellitus	History of type 1 or type 2 diabetes (documented diagnosis or HbA1c ≥ 6.5%)	0.0%	Not applicable
Hypertension	Documented diagnosis of hypertension or use of antihypertensive medication	0.0%	Not applicable
Malignancy	Active or historical solid or hematologic malignancy	0.0%	Not applicable
Smoking Status	Current or former smoker (any use within 6 months pre-op)	0.0%	Not applicable
Chronic Kidney Disease	Documented CKD stage ≥3 (eGFR <60 mL/min/1.73m^2)	0.0%	Not applicable
Chronic Obstructive Pulmonary Disease	Documented diagnosis based on spirometry or medical history	0.0%	Not applicable
ASA Score	American Society of Anesthesiologists physical status classification (I–IV)	0.0%	Not applicable
Emergency Surgery	Operation performed within 24 hours of admission	0.0%	Not applicable
Open Fracture	Any skin breach associated with fracture (Gustilo–Anderson classification I–III)	0.0%	Not applicable

Tourniquet Use	Application of pneumatic tourniquet during surgery	0.0%	Not applicable
Time to Surgery (hours)	Time from admission to incision, in hours	0.0%	Not applicable
Operative Time (minutes)	Time from incision to closure, in minutes	0.0%	Not applicable
Minimally Invasive Technique	Surgery performed through percutaneous or limited approach	0.0%	Not applicable
External Fixation	Use of external fixation device during definitive stabilization	0.0%	Not applicable
Flap Coverage	Soft tissue coverage procedure performed during index operation	0.0%	Not applicable
Bone Grafting	Use of autologous or allograft bone graft material	0.0%	Not applicable
Drain Insertion	Placement of any surgical drain (active or passive)	0.0%	Not applicable
Reoperation	Return to operating room within index hospitalization	0.0%	Not applicable
Blood Transfusion	Receipt of red blood cell units during or within 24 hours of surgery	0.0%	Not applicable
Estimated Blood Loss (mL)	Estimated intraoperative blood loss in milliliters	1.1%	Predictive Mean Matching
Length of Hospital Stay (days)	Total inpatient days from admission to discharge	0.0%	Not applicable
Hemoglobin (g/dL)	Preoperative hemoglobin within 72 h prior to surgery	0.5%	Predictive Mean Matching
Red Blood Cell Count ($\times 10^6/\mu L$)	Preoperative RBC count within 72 h prior to surgery	0.9%	Predictive Mean Matching
White Blood Cell Count ($\times 10^9/L$)	Preoperative WBC count within 72 h prior to surgery	0.8%	Predictive Mean Matching
Neutrophil Count ($\times 10^9/L$)	Preoperative neutrophil count within 72 h prior to surgery	0.8%	Predictive Mean Matching

Lymphocyte Count ($\times 10^9$/L)	Preoperative lymphocyte count within 72 h prior to surgery	0.8%	Predictive Mean Matching
Platelet Count ($\times 10^3$/μL)	Preoperative platelet count within 72 h prior to surgery	0.9%	Predictive Mean Matching
Prothrombin Time (sec)	Preoperative PT from lab within 72 h prior to surgery	1.2%	Predictive Mean Matching
APTT (sec)	Preoperative activated partial thromboplastin time within 72 h prior to surgery	1.6%	Predictive Mean Matching
Albumin (g/dL)	Serum albumin within 72 h preoperatively	2.3%	Predictive Mean Matching
Glucose (mg/dL)	Serum glucose within 72 h before surgery	1.7%	Predictive Mean Matching
D-Dimer (mg/L)	D-dimer within 72 h before surgery	3.1%	Predictive Mean Matching
Preoperative CRP (mg/L)	C-reactive protein measured within 72 h before surgery	1.9%	Predictive Mean Matching
Postoperative CRP (mg/L)	C-reactive protein measured within 24–72 h after surgery	2.7%	Predictive Mean Matching

Supplementary Table 2. Feature Selection Thresholds and Multicollinearity Handling

**Feature Selection Strategy**	**Retention Threshold/Rule**	**Multicollinearity Handling Approach**
Bootstrap Inclusion Frequency	Variables selected in ≥60% of 1000 bootstrap replications were retained in the final model.	Variance Inflation Factor (VIF) calculated; variables with VIF > 10 were excluded. Final model showed condition number = 6.4.
LASSO	Optimal lambda (λ) chosen via 10-fold cross-validation (minimum deviance criterion).	Not required due to inherent regularization in LASSO.
Univariate Filtering (p < 0.20)	All variables with p < 0.20 in univariate comparisons were retained.	VIFs > 10 flagged, but not excluded unless causing complete separation.
Stepwise Selection (Backward AIC)	Retained variables with backward elimination minimizing AIC.	VIF calculated during final model assembly; no variable exceeded VIF > 10.
Boruta Algorithm	All variables classified as "Confirmed" by the Boruta algorithm were selected.	Ridge penalization applied post-selection; additionally, VIF screening removed 2 highly collinear variables (VIF > 15).
Recursive Feature Elimination (RFE)	Variables recursively selected using 10-fold cross-validated AUC optimization.	VIFs evaluated post-selection; all retained variables showed acceptable multicollinearity (VIF < 8).
